# Interparental conflict and adolescents’ suicidal ideation: life satisfaction as a mediator and teacher support as a moderator

**DOI:** 10.3389/fpubh.2025.1637974

**Published:** 2025-10-17

**Authors:** Lu Xu, Zhuang She, Baohua Xu

**Affiliations:** ^1^Yichang Special Care Hospital, Yichang, China; ^2^Yichang Clinical Research Center for Mental Disorders, Yichang, China; ^3^Department of Psychology, Nanjing University, Nanjing, China

**Keywords:** interparental conflict, life satisfaction, teacher support, suicidal ideation, adolescents

## Abstract

**Background:**

Suicidal ideation is the most significant risk factor for suicide, and suicide is the third leading cause of death among people aged 15 to 19 years. Interparental conflict has been shown to be associated with adolescents’ suicidal ideation, but the reasons for this association remain underexplored. We investigated whether adolescents’ life satisfaction accounts for this relationship, and whether perceived teacher support moderates the mediation process.

**Methods:**

A total of 649 Chinese adolescents (52% girls; mean age = 15.59 years, *SD* = 0.70) completed anonymous questionnaires in their classroom to assess interparental conflict, life satisfaction, teacher support, and suicidal ideation. The data were analyzed using SPSS 26.0 software.

**Results:**

The mediation analysis showed that a significant indirect relationship between interparental conflict and suicidal ideation, mediated by life satisfaction (*β* = 0.02, 95% CI [0.01, 0.04]). The moderation analysis revealed that teacher support moderated the relationship between life satisfaction and suicidal ideation (*β* = −0.03, *p* < 0.01). The relationship between life satisfaction and suicidal ideation was significant for adolescents who perceived high teacher support (*β* = −0.08, 95% CI [−0.12, −0.04]) but not for those who perceived low teacher support (*β* = −0.01, 95% CI [−0.05, 0.03]).

**Discussion:**

The study suggest that life satisfaction and teacher support are important protective factors for adolescent suicidal ideation. Life satisfaction was associated with less suicidal ideation for adolescents with high rather than low teacher support. These findings point to the importance of considering school, family, and individual factors concurrently when developing programs to prevent and reduce adolescents’ suicidal ideation.

## Introduction

Suicide is the third most common cause of death among young people aged 15 to 29 (1). The issue of adolescent suicide in China is still alarming (2). Adolescence is characterized by asynchronous brain development, whereby the subcortical limbic system exhibits more accelerated maturation compared to the orbitofrontal cortex. This disproportional development can undermine rational decision-making abilities and potentially elevate the risk of suicidal behavior, driven by heightened sensation seeking and emotional dysregulation (3). Adolescence itself is a risk factor for suicidal ideation and behavior (4). Understanding the risk and protective factors associated with adolescent suicide is essential for developing effective prevention and intervention strategies.

Suicidal ideation encompasses the contemplation of ending one’s life, even if such thoughts do not invariably culminate in an actual suicide. It is an early indicator of future suicidal actions and it is also the most sensitive and significant risk factor contributing to the final behavior (5, 6). Thus, the identification of predictors of ideation might lead to a better understanding of suicide risk, and provide adolescents with the help they need (7, 8).

The influence of interparental conflict on adolescent suicidal ideation has garnered notable attention [e.g., (5, 9, 10)]. Given the significant emphasis on collectivism and family relations in Chinese culture, the ramifications of interparental conflict on Chinese adolescents may be particularly severe (11). According to emotional security theory (EST), exposure to destructive forms of interparental conflict (e.g., hostile; aggressive; unresolved) may endanger the child’s emotional security, potentially resulting in adjustment problems (12). Several empirical studies have shown a significant relationship between interparental conflict and adolescents’ suicidal ideation (5, 9, 10). For instance, Ai et al. (5) found that interparental conflict posed a significant risk for suicidal ideation and suicide attempts among adolescents. Interparental conflict was shown in one study to indirectly predict adolescents’ suicidal ideation via coping-approach strategies, presence of meaning, and their joint serial effects (10).

Although there is evidence of a direct relationship between interparental conflict and adolescent suicidal ideation, the mediating effects (i.e., the pathways through which interparental conflict influences suicidal ideation) and the moderating factors (i.e., the factors that affect the strength or direction of this relationship) in this association remain largely unexplored. Most suicidal ideation research focuses on risk factors, and the exploration of protective factors is relatively rare (13). Moreover, there is a lack of research on how external environmental resources interact with individual internal resources to predict suicidal ideation. Addressing these gaps is crucial for understanding the etiology of adolescent suicidal ideation and for creating prevention strategies. In this study, we addressed two key questions: first, whether the relationship between interparental conflict and adolescent suicidal ideation can be explained in part by life satisfaction; second, whether this indirect association via life satisfaction is moderated by teacher support.

### The mediating role of life satisfaction

There has been considerable research attention to negative psychological factors (e.g., substance abuse; depression) as predictors of adolescent suicidal ideation (14, 15). However, it is also crucial to include positive psychological factors as predictors of lower suicidal ideation (6). Psychologists are interested not just in pathology but also in the well-being of individuals (16). Life satisfaction, an important element of quality of life and subjective well-being, encapsulates the cognitive appraisal of one’s overall quality of life or of specific life domains (17). Life satisfaction, while generally stable over time, exhibits sensitivity to changes in life circumstances (18). We thus used life satisfaction as the indicator of adolescents’ well-being in the current study.

According to the emotional security theory [EST; (12)], interparental conflict leads to adolescents’ negative internal representations of their well-being, thereby increasing their risk of maladaptive outcomes (12). Likewise, the conservation of resources theory (19) posits that individuals strive to retain, protect, and build resources, and that negative stress events decrease an individual’s mental health due to the mediating effects of loss of resources (e.g., lower personal sense of well-being; lower sense of control) loss. In other words, lower life satisfaction may mediate the relationship between interparental conflict and adolescents’ suicidal ideation.

Consistent with these theoretical frameworks, some empirical evidence has demonstrated the mediating effect of life satisfaction in the association between negative environments and adolescent maladaptive outcomes (20, 21). Specifically, Moksnes et al. (21) found that school performance pressure was a positive predictor of Norwegian adolescents’ depressive symptoms, and that this association was mediated by life satisfaction. In a study of 3,522 adolescents in China, Chang et al. (20) found that life satisfaction partially mediated the relationship between cyberbullying victimization and suicidal ideation. Although not yet tested, it is reasonable to expect that life satisfaction will also mediate the association between interparental conflict and adolescent suicidal ideation.

We will review prior research findings to substantiate our argument. First, adolescents who perceive more interparental conflict are more likely to report lower life satisfaction. High cohesion among family members is associated with higher parental concern for their children, fostering a harmonious family atmosphere and with higher life satisfaction among Chinese young adults (22). Similarly, Zhu et al. (23) found that the frequency of interparental conflict negatively predicted the life satisfaction of Chinese adolescents. Second, adolescents with lower levels of life satisfaction may relate to more suicidal ideation. Several studies have indicated that low life satisfaction serves as a significant predictor of suicidal ideation among adolescents (6, 20). For instance, Morales-Vives and Dueñas (6) found that, potentially due to the stress generated by the physical and emotional transitions during adolescence, Spanish adolescents reported lower life satisfaction compared to younger children, which in turn was associated with more suicidal ideation.

Therefore, building on the emotional security theory (12), the conservation of resources theory (19) and the extant literature, we propose that life satisfaction mediates the relationship between interparental conflict and adolescent suicidal ideation.

### The moderating role of teacher support

There is variability in how youth are affected by interparental conflict, and further research is needed to identify protective factors that can mitigate the negative impacts of such conflict on adolescents (24). The social support resource theory (25) posits that social support, as a significant external resource, can enhance individuals’ internal resources, exhibiting a “the rich get richer” phenomenon; this mutual enrichment can improve individuals’ capacity to cope with risks. Similarly, according to the protective-protective model (26), teacher support is a crucial external protective factor for adolescents’ healthy development; it can enhance the effect of another promotive factor in producing a certain outcome, and the interaction goes beyond the alone effects of either factor. Based on these theories, we infer that the association between life satisfaction and adolescent suicidal ideation may be moderated by teacher support as an protective factor. The advantageous impact of life satisfaction may be more pronounced in adolescents who receive high levels of teacher support compared to those with low levels of support.

We have the following reasons for examining teacher support as a moderator: (a) Several studies have established teacher support as an important protective factor against adolescent suicidal ideation (27, 28); (b) Given the substantial amount of time that youth spend in educational settings, teachers are uniquely positioned to recognize signs of suicide risk and take appropriate prevention measures (29); (c) Teachers’ attitudes can be changed through suicide intervention programs (30), and as significant others who frequently interact with adolescents, they have a crucial impact on their mental health (31).

Although relatively limited, existing research has provided evidence on the moderating effect of social support in the direct relationship between promotive factors and suicidal ideation [e.g., (32, 33)]. Specifically, Zhang et al. (33) found that for Chinese older adults with higher support from nursing, resilience was significantly associated with lower suicidal ideation. The association between emotional intelligence and suicidal ideation was negatively significant among Spanish adolescents with high family support (32). Compared to peer support, teacher support is a more significant moderator and protective factor for Chinese adolescent suicidal inclination (28). Thus, we anticipate that the association between life satisfaction and suicidal ideation is stronger among adolescents with high rather than low teacher support.

### Overview of the study and hypotheses

**Figure 1:**
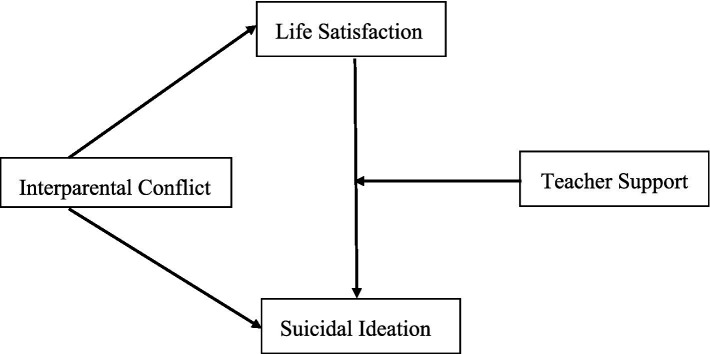
The proposed moderated mediation model.

In the present study we tested a moderated mediation model to elucidate the association between adolescents’ reports of interparental conflict and their own suicidal ideation. Adolescents’ self-reported life satisfaction was tested as the mediator. Adolescents’ perception of teacher support was tested as a moderator that would enhance the second mediation link (the association between life satisfaction and suicidal ideation). Hypothesis 1: Life satisfaction will mediate the relationship between interparental conflict and adolescent suicidal ideation. Hypothesis 2: Teacher support will moderate the second link in the indirect pathway; that is, the association between life satisfaction and suicidal ideation is stronger among adolescents with high rather than low teacher support. Figure 1 illustrates the proposed research model.

## Methods

### Participants and procedures

This study was approved by the research ethics committee of our institution (approval number AF/SC-05/02.1). Convenience sampling was used to recruit participants from two high schools in Hubei Province, China. The school administrators approved the study and informed consent was obtained from the participants prior to data collection. Assurances were provided regarding the anonymity of their responses. The questionnaires were administered in students’ classrooms by trained research assistants, who followed standardized scripts and procedural manuals to ensure accurateness in data collection. Participants who finished the full survey received a gift of five yuan (about US $0.80).

The sample constituted 649 students, with 313 boys and 336 girls. The average age of the participants was 15.59 years (*SD* = 0.70, range = 14–18). About half (52%) were in Grade 10, and about half (48%) were in Grade 11.

### Measures

#### Interparental conflict

The Chinese version of the Children’s Perception of Marital Conflict Scale (34) was employed. This scale, originally in English (35) and extensively referenced in the literature, comprises 15 items designed to assess interparental conflict across three dimensions: intensity, frequency, and resolution (the latter being reverse coded to reflect effective disagreement resolution without anger or aggression). An example item is “My parents fight when they have a quarrel.” Adolescents rated the veracity of each item on a 4-point Likert scale from 1 = Not True, 2 = A Little Bit True, 3 = Sort of True, 4 = True. The average of the 15 item scores represented the degree of interparental conflict, with higher scores indicating more conflict. The scale demonstrated excellent reliability in this study (*α* = 0.90).

#### Life satisfaction

The Satisfaction with Life Scale [SWLS; (36)] is a 5-item instrument designed to assess global life satisfaction. Respondents rate each item on a 7-point Likert scale, ranging from 1 (strongly disagree) to 7 (strongly agree). An example item is “In most ways my life is close to my ideal.” This questionnaire has been utilized in many prior studies and is recognized for its reliability and validity in evaluating Chinese adolescents’ life satisfaction (37). A higher cumulative score on the SWLS indicates a greater level of life satisfaction. In the present study, Cronbach’s α for the scale was 0.75.

#### Teacher support

The Chinese version of Perceived Social Support Scale (38) was utilized in this study. Perceived social support from family, friends, and teacher are measured separately, and we only used the four items on the teacher support subscale. A sample item is “My teacher helps me when I have trouble.” Items are rated on a 7-point Likert scale (1 = strongly disagree, 7 = strongly agree). Cronbach’s alpha for the present sample was good (α = 0.84).

#### Suicidal ideation

We used a single item to measure adolescents’ suicidal ideation: “During the last 6 months, I have thought about killing myself.” Respondents rated this item on a three-point Likert scale ranging from 0 (never) to 2 (frequently). The item we used has been used in previous studies on adolescents attending high school in China [e.g., (5, 39)].

#### Control variables

Chang et al. (2) found that the prevalence of suicidal ideation were significantly higher among Chinese girls than among boys due to the differences in susceptibility to psychosocial distress and emotional issues. The prevalence rates of suicidal ideation significantly increased with age in Chinese adolescents (40). Moreover, emerging evidence suggests that family financial hardships may heighten adolescents’ risk for suicide (41). Therefore, we included gender (dummy coded as 0 for girl and 1 for boy), age (as a continuous variable) and family financial stress as covariates in the analyses. The Family Financial Stress Scale was compiled by Chinese scholars Wang et al. (42). It includes 5 items, such as “My family does not have any remaining money for family entertainment.” Participants are required to report the frequency of economic pressure in their families over the past 12 months. A 4-point scale is used, ranging from “never” (1 point) to “always” (4 points). The average score of all items is calculated, with higher scores indicating greater family financial stress. In this study, Cronbach’s alpha for the scale was 0.83.

### Statistical analysis

All statistical analyses were performed using SPSS 26.0 software (IBM Corp, Armonk, NY, United States). First, descriptive statistics were generated for the primary study variables, followed by Pearson correlation analysis to examine the relationships among variables. Subsequently, the PROCESS macro (43), a widely used tool for examining complex models involving moderation and mediation effects, was employed to test the moderated mediation model with 5,000 bias-corrected bootstrap samples. These bootstrap samples were used to estimate 95% confidence intervals (CIs), with an effect considered statistically significant if the 95% CI did not include zero. Specifically, Model 4 of the macro was used to test a mediation model with life satisfaction as the mediator. Then, Model 14 of the macro was employed to examine an integrated model with life satisfaction as the mediator and teacher support as the moderator.

## Results

### Variance inflation factor and common method Bias test

Prior to main analyses, we conducted multicollinearity diagnostics. Variance Inflation Factors (VIF) for all predictor variables were well below the conventional threshold of 5, indicating no significant multicollinearity issues. Since the data were collected via self-report, concerns about common method bias may arise. Harman’s single-factor test revealed that there were 7 factors with eigenvalues greater than 1, and the first factor accounted for 21.9% of the variance—below the critical threshold of 40%. Thus, the threat of common method bias appears negligible.

### Preliminary analyses

Table 1 presents univariate and bivariate statistics, including means and standard deviations, for all study variables. Within the total sample, 21.9% (*n* = 142) of participants reported experiencing suicidal ideation in the preceding 6 months. Specifically, 71.8% (*n* = 507) of participants gave a rating of 0 (never), 20.2% (*n* = 131) of participants gave a rating of 1 (occasionally), and 1.7% (*n* = 11) of participants gave a rating of 2 (frequently). Interparental conflict was positively correlated with suicidal ideation. Both life satisfaction and teacher support were negatively correlated with suicidal ideation. Teacher support was positively correlated with life satisfaction. Results were as expected.

**Table 1:** Descriptive statistics and correlations among all study variables.

Variable	*M*	*SD*	1	2	3	4	5	6	7
1. Gender^a^	0.48	0.50	—						
2. Age in years	15.59	0.70	0.21***	—					
3. Family financial stress	1.99	0.58	0.09*	0.05	—				
4. Interparental conflict	1.39	0.47	−0.03	0.03	0.14***	—			
5. Life satisfaction	3.92	1.20	0.06	0.01	−0.17***	−0.20***	—		
6. Teacher support	3.99	1.30	−0.10*	0.02	−0.02	−0.06	0.27***	—	
7. Suicidal ideation	1.51	0.31	0.02	−0.01	0.04	0.15***	−0.16***	−0.13***	—

*N* = 649. ^a^Gender was dummy coded as 0 = girls and 1 = boys. *p < 0.05. **p < 0.01. ***p < 0.001.

### Mediation effect of life satisfaction

Controlling for gender, age and family financial stress, the 95% confidence interval derived via bootstrapping substantiated a significant indirect relationship between interparental conflict and suicidal ideation, mediated by life satisfaction (*β* = 0.02, 95% CI [0.01, 0.04]). Specifically, interparental conflict demonstrated a significant direct relationship with suicidal ideation (*β* = 0.12, *p <* 0.001). Interparental conflict was negatively correlated with life satisfaction (*β* = −0.37, *p* < 0.001), and life satisfaction negatively predicted suicidal ideation (*β* = −0.05, *p <* 0.001). These results supported *H_1_*.

### Moderated mediation effects

The results of the moderated mediation model analysis are reported in two parts. First we report the moderation effects. Second, we report the mediation effects (conditional indirect effects). Table 2 shows that the interaction effect of life satisfaction and teacher support on suicidal ideation was significant after controlling for gender, age, and family financial stress, *β* = −0.03, *p* < 0.01. Thus, teacher support moderated the association between life satisfaction and suicidal ideation.

**Table 2:** Moderated mediation model test.

	*β*	*SE*	*t*	*p*	95% CI
Outcome: Sucidal ideation					
Gender^a^	0.01^a^	0.04	0.40	0.69	[−0.06, 0.09]
Age in years	−0.00	0.03	−0.15	0.88	[−0.06, 0.05]
Family financial stress	−0.00	0.04	−0.01	0.99	[−0.08, 0.08]
Interparental conflict	0.10**	0.03	3.01	< 0.01	[0.03, 0.16]
Life satisfaction (LS)	−0.05**	0.02	−2.96	< 0.01	[−0.08, −0.02]
Teacher support (TS)	−0.03**	0.01	−2.28	< 0.01	[−0.06, −0.00]
LS × TS	−0.03**	0.01	−2.60	< 0.01	[−0.05, −0.01]
Outcome: Life satisfaction					
Gender^a^	0.15^a^	0.09	1.55	0.12	[−0.04, 0.33]
Age in years	0.02	0.07	0.27	0.79	[−0.11, 0.15]
Family financial stress	−0.38***	0.10	−3.84	< 0.001	[−0.57, −0.18]
Interparental conflict	−0.37***	0.08	−4.69	< 0.001	[−0.53, −0.22]

*N =* 649. All continuous predictors were mean centered prior to analysis. ^a^Gender was dummy coded such that 0 = girls and 1 = boys. *p < 0.05. **p < 0.01. ***p < 0.001.

**Figure 2:**
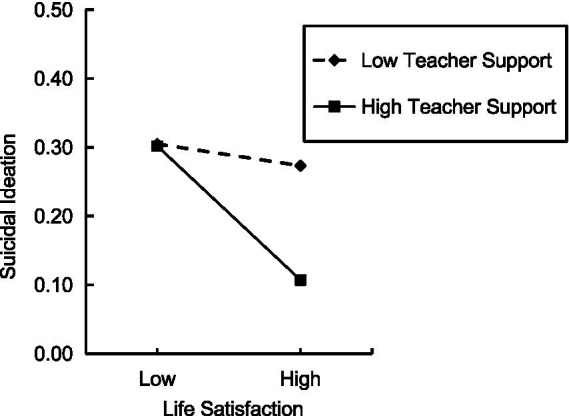
The association between life satisfaction and suicidal ideation at different levels of teacher support. Teacher support levels were created based on the *M* and *SD* of the teacher support scores: Low = *M* − 1 *SD*; High = *M* + 1 *SD*.

Table 3 shows the mediation effects at +1 *SD*, *M*, and −1 *SD* of teacher support scores. All slopes were negative. However, the 95% confidence interval included zero (was non-significant) when the teacher support score was one standard deviation below the mean, and the 95% confidence interval excluded zero (was significant) when the teacher support score was at the mean and at one standard deviation above the mean. Specifically, the indirect effect of interparental conflict on suicidal ideation through life satisfaction was significant for adolescents with a higher level of teacher support (+1 *SD*; *β* = −0.08, 95% CI [−0.12, −0.04]) and an average level of support (*M*; *β* = −0.05, 95% CI [−0.07, −0.02]), but was non-significant for adolescents with a lower level of teacher support (−1 *SD*; *β* = −0.01, 95% CI [−0.05, 0.03]). These results supported *H_2_* for purposes of illustration. Figure 2 depicts this interaction when teacher support scores were low (*M–* 1 *SD*), average (*M*) and high (*M* + 1 *SD*).

**Table 3:** Significance testing of the moderated mediation effect.

Teacher support	*β*	Boot *SE*	95% CI
Low	−0.01	0.02	[−0.05, 0.03]
Average	−0.05	0.02	[−0.07, −0.02]
High	−0.08	0.02	[−0.12, −0.04]

*N* = 649. Teacher support levels were based on the M and SD of the teacher support scores: Low = M − 1 SD; Average = M; High = M + 1 SD. SE = standard error. 95% CI = 95% bootstrapped confidence interval; a CI that does not contain 0 is statistically significant.

## Discussion

We developed and evaluated a moderated mediation model based on an integration of existing theories (i.e., EST, the conservation of resources theory, and the protective-protective model) to elucidate the interconnections between suicidal ideation and various influencing factors, including a family factor (interparental conflict), a school factor (teacher support), and an individual-level factor (life satisfaction). Our findings indicated that life satisfaction mediated the relationship between interparental conflict and suicidal ideation, with this indirect effect being moderated by the presence of teacher support. The mediating effect was significant among adolescents with a high or average level of teacher support, but not among adolescents with a low level of teacher support.

In our sample, 21.9% (*n* = 142) of adolescents reported suicidal ideation in the past 6 months; 1.7% (*n* = 11) reported frequent thoughts, and 20.2% (*n* = 131) reported occasional thoughts. These prevalence estimates are higher than those reported in prior studies of Chinese adolescents (5, 39), a difference that may reflect effects of the COVID-19 pandemic. Recent evidence documents worsening adolescent mental health and increases in suicidal ideation and self-harm during and following the COVID-19 pandemic (44). These results emphasize the heightened severity of adolescent suicidal risk in the pandemic context and the imperative for early detection and intervention in the post-pandemic period.

Adolescent life satisfaction mediated the relationship between interparental conflict and suicidal ideation. This finding is congruent with EST (12), the conservation of resources theory (19) and previous research (18, 20, 23). Adolescence is important for the development of internalizing and externalizing problems, and individuals at this stage are particularly sensitive to psychosocial stressors (45). Among the various family stressors, interparental conflict poses the most severe stress on adolescents (18). Interparental conflict can deplete adolescents’ resources, such as parental emotional availability, sensitivity, belongingness, and sense of well-being, and the loss of these resources ultimately impacts mental health (12, 19). Adolescents’ need for belongingness might not be fulfilled in an environment with interparental conflict, and intrinsic motivation diminishes (46). Low intrinsic motivation might in turn reduce creativity and life satisfaction, ultimately exerting a negative impact on mental health (46).

Furthermore, adolescence is a transformative stage of life, characterized by numerous physical, cognitive, and psychosocial changes, and is important for the development of self-identity (47). For adolescents who have developed the ability to self-evaluate, prolonged exposure to high levels of interparental conflict may be closely associated with negative internal self-evaluations and experiences (48). Such negative self-evaluation is related to lower life satisfaction among adolescents (49). Shneidman (50) asserted that low life satisfaction can elevate psychache, and when this psychache exceeds an individual’s tolerance it may lead to suicide as a means of escaping the distress.

Our findings substantiate the assumption that teacher support influences the indirect relationship between interparental conflict and adolescent suicidal ideation. Consistent with the protective-protective model (26), we found that life satisfaction was associated with less suicidal ideation for adolescents with high rather than low teacher support. Person-centered theory (51) can also explain our findings. Rogers asserted that a supportive interpersonal environment (e.g., understanding and support from others) helps individuals in more effectively identifying and utilizing their own resources (e.g., life satisfaction) to cope with various psychological problems (51). In other words, life satisfaction, serving as an individual resource, may have its protective function strengthened due to high levels of teacher support. A good teacher support system can provide favorable environmental conditions for the development of adolescents’ life satisfaction (52, 53).

In addition, Pluess and Belsky (54) proposed the concept of vantage sensitivity, which refers to some individuals benefiting more than others from a positive environmental experience or exposure. This concept is consistent with evidence that American high school students who reported higher levels of life satisfaction benefited more from numerous positive factors, such as higher social support (55). Hence, it is possible that adolescents with higher life satisfaction are more likely to benefit from teacher support, which is associated with less suicidal ideation among adolescents (28). Because adolescents spend most of their time at school (56), teachers play a crucial role in preventing adolescent suicide (30).

However, person-centered theory (51) suggests that low-level interpersonal support greatly restricts individuals’ potential to utilize their own resources to cope with risks. Similarly, social support resource theory (25) indicates that resources tend to enrich each other, but a lack of social support can lead to a cycle of resource loss, thereby reducing an individual’s coping ability. These may explain why the negative correlation between life satisfaction and suicidal ideation was not significant among adolescents with a low level of teacher support. Fergus and Zimmerman (26) pointed out that support from a nonparental adult mentor, as a protective resource for adolescents, the protective effect may not significant when lack of it (26). This finding is similar to the results of a recent study found that the relationship between emotional intelligence and suicidal ideation was significant for Spanish adolescents with medium or high family support but not significant for those with low family support (32).

Teachers can offer crucial intervention and support to adolescents experiencing suicidal ideation (57). Indeed, 36.4% of teachers have reported instances of students (from kindergarten to twelfth grade) revealing suicidal ideation to them (30). Despite this critical role, many teachers may feel reluctant to intervene due to the significant burden and responsibility associated with it (30). Following a student’s suicide, over one-third of primary and secondary school teachers reported a decline in their professional confidence and a need for more support (58). Therefore, it is imperative to deepen our understanding of the importance teachers attribute to their role and give teachers support they need in suicide prevention.

It is noteworthy that the protective role of teacher support against student suicidal ideation may manifest culturally specific functions. In Chinese collectivist culture, relational dependence and intimacy in teacher-student relationships are more socially acceptable and perceived as characteristics of better social adaptation; whereas in Western countries, students’ relational dependence on teachers may conflict with cultural values emphasizing autonomy and independence, thus being regarded as behaviors to be avoided (59). Chen et al. (60) demonstrated that compared to Dutch students, Chinese students exhibit stronger desire to seek validation and assistance from teachers when experiencing distress, with the safe haven function in teacher-student relationships being more prominent in collectivist cultural contexts.

### Limitations and future directions

Several limitations warrant consideration in interpreting the results. First, the cross-sectional design is a major limitation of this study. Future research should employ longitudinal tracking or experimental interventions (for example, programs aimed at improving life satisfaction) to assess causal impacts on adolescent suicidal ideation. Furthermore, adolescents in early, middle, and late stages differ meaningfully in life satisfaction, social support, and emotion-regulation capacities (53, 61). Therefore, the effect of interparental conflict on suicidal ideation, the mediating role of life satisfaction, and the moderating influence of teacher support may be age-dependent. We recommend that future studies adopt longitudinal, stage-specific designs to examine developmental variation in these mediation and moderation processes, which would enable the development of age-tailored prevention and intervention measures.

Second, adolescents are mostly engaged with home and school settings and overall assessments of life satisfaction may obscure differences in adolescents’ relationships with their family and school (62). Chang et al. (20) found that satisfaction with family life exerted the greatest indirect effect in the relationship between cyberbullying and suicidal ideation, followed by satisfaction with peers and academic performance. Future research is recommended to further compare the potential differential effects of various dimensions of life satisfaction (e.g., family life satisfaction, campus life satisfaction) in the association between interparental conflict and suicidal ideation.

Third, this study was conducted in a school-based Chinese sample; therefore, the conclusions of this study may primarily apply to non-clinical adolescent populations in similar cultural contexts. After all, the collectivist culture in China and the independent culture in the West may lead to differences in students’ attitudes and responses toward teacher support (59, 60). Future research should examine the current model in clinical samples and across diverse cultural backgrounds, while also taking into account cultural variations in teacher-student interactions to enhance the applicability and representativeness of the findings.

Fourth, this study concentrates on the relationship between interparental conflict and suicidal ideation; however, other factors may also constitute significant risk factors for suicidal ideation, such as suicide contagion and cyberbullying. Recent research shows that suicide contagion substantially increases the likelihood of suicidal ideation among adolescents (63). Additionally, Maurya et al. (64) found that adolescent cyberbullying victims were at higher risk of suicidal ideation. Consequently, future studies could control for these variables and further examine the relationship between interparental conflict, life satisfaction, and suicidal ideation.

Finally, this study used a single-item measure to assess the frequency of suicidal ideation. Although empirical findings support the validity of single-item assessments for suicidal ideation frequency (65), multi-item instruments can more comprehensively capture the severity, frequency, and qualitative features of suicidal ideation. We recommend future studies use validated multi-item scales [e.g., the Beck Scale for Suicidal Ideation; (66)] to obtain multidimensional and more nuanced measures of suicidal ideation.

### Implications

Our findings make several contributions to the literature and have practical implications. First, the results highlight teacher support as a protective factor in predicting suicidal ideation. This extension to the literature is consistent with the call to identify positive psychological factors that are associated with lower suicide risk, not only negative psychological factors that predict higher risk (6, 13).

Second, this study confirms the importance of interactions between multiple ecosystems. The ecological systems theory (67) posits that the mental health of adolescents is influenced by the combined effects of microsystems (such as family and school) and individual characteristics. These factors do not exist in isolation but are intertwined and mutually influential. Suicide is caused by the complex interplay of multiple factors, and prevention strategies should consider broader personal and ecological aspects (68). This study offers a nuanced perspective that integrates personal, family, and school contexts. This comprehensive perspective facilitates a thorough understanding of the factors influencing adolescent suicidal ideation.

Third, our findings suggest that mitigating interparental conflict may contribute to a decrease in adolescents’ suicidal ideation. Interventions that include a focus on suicidal ideation (such as interventions for depression) could include a component for youth and a component for parents. Grych et al. (69) found that self-blame mediated the association between interparental conflict and adolescents’ internalizing problems. Cognitive therapy could help youth to change their assumptions about being to blame for their parents’ destructive conflict or being responsible to fix the problem. Parents could be helped to reduce destructive forms of conflict and increase conflict resolution. Adolescents’ ability to regulate emotions can potentially be enhanced by witnessing constructive conflicts between their parents (12). Encouraging couples to reassess conflicts with their partners is also a concise yet effective approach that can reduce conflicts and enhance marital quality (70).

Fourth, our research also points to life satisfaction as a possible mechanism through which interparental conflict is correlated with adolescent suicidal ideation. This information can be helpful for practitioners in conducting individual therapy with adolescents and in designing targeted interventions that include a component related to life satisfaction. Numerous environmental, familial, and social factors contribute to youth life satisfaction. These include maintaining a healthy lifestyle, achieving good physical health, engaging in regular exercise, participating in sports, living in a secure neighborhood, infrequent relocations, good parental relationships and social support (71).

Finally, it should be noted that in our study, life satisfaction predicted lower suicidal ideation more strongly for students with high teacher support than for those with low teacher support. This suggests a need for teacher training to provide support for adolescents. Considering that many teachers feel reluctant to intervene, training programs for teachers in adolescent suicide prevention should focus on enhancing teachers’ self-efficacy and positive outcome expectations (30). Together, the results suggest that suicide prevention programs should focus not only on the individual adolescent but also on the school and family.

## Data Availability

The original contributions presented in the study are included in the article/supplementary material, further inquiries can be directed to the corresponding author.
