# Correction: Interparental conflict and adolescents' suicidal ideation: life satisfaction as a mediator and teacher support as a moderator

**DOI:** 10.3389/fpubh.2025.1728911

**Published:** 2025-10-27

**Authors:** Frontiers Production Office

**Affiliations:** Frontiers Media SA, Lausanne, Switzerland

**Keywords:** interparental conflict, life satisfaction, teacher support, suicidal ideation, adolescents

In the **Abstract**, corrections to the values in the **Results** paragraph of the abstract were not implemented. This has been corrected to read:

“**Results:** The mediation analysis showed that a significant indirect relationship between interparental conflict and suicidal ideation, mediated by life satisfaction (β = 0.02, 95% CI [0.01, 0.04]). The moderation analysis revealed that teacher support moderated the relationship between life satisfaction and suicidal ideation (β = −0.03, *p* < 0.01). The relationship between life satisfaction and suicidal ideation was significant for adolescents who perceived high teacher support (β = −0.08, 95% CI [−0.12, −0.04]) but not for those who perceived low teacher support (β = −0.01, 95% CI [−0.05, 0.03]).”

The original version of this article has been updated.

